# Exchange controlled triplet fusion in metal–organic frameworks

**DOI:** 10.1038/s41563-022-01368-1

**Published:** 2022-10-06

**Authors:** Dong-Gwang Ha, Ruomeng Wan, Changhae Andrew Kim, Ting-An Lin, Luming Yang, Troy Van Voorhis, Marc A. Baldo, Mircea Dincă

**Affiliations:** 1 Department of Chemistry, Massachusetts Institute of Technology, Cambridge, MA USA; 2 Department of Electrical Engineering and Computer Science, Massachusetts Institute of Technology, Cambridge, MA USA

**Keywords:** Organic-inorganic nanostructures, Optical materials and structures

## Abstract

Triplet-fusion-based photon upconversion holds promise for a wide range of applications, from photovoltaics to bioimaging. The efficiency of triplet fusion, however, is fundamentally limited in conventional molecular and polymeric systems by its spin dependence. Here, we show that the inherent tailorability of metal–organic frameworks (MOFs), combined with their highly porous but ordered structure, minimizes intertriplet exchange coupling and engineers effective spin mixing between singlet and quintet triplet–triplet pair states. We demonstrate singlet–quintet coupling in a pyrene-based MOF, NU-1000. An anomalous magnetic field effect is observed from NU-1000 corresponding to an induced resonance between singlet and quintet states that yields an increased fusion rate at room temperature under a relatively low applied magnetic field of 0.14 T. Our results suggest that MOFs offer particular promise for engineering the spin dynamics of multiexcitonic processes and improving their upconversion performance.

## Abstract (web summary)

Triplet-fusion-based photon upconversion is promising for photovoltaic or bioimaging applications, but its efficiency is limited by triplet fusion spin dependence. Here, the authors tailor spin dynamics by engineering the crystal structure with metal–organic frameworks to enable effective spin mixing between singlet and quintet triplet–triplet pair states.

## Main

Singlet exciton fission and the reverse process of triplet–triplet (TT) fusion are unique spin-dependent phenomena in organic materials that have attracted interest in the context of a diverse range of applications, including photovoltaics^1^, biomedical imaging^2^, photochemical reactions^3^, organic light-emitting devices^4^ and quantum information^5,6^. In contrast with conventional nonlinear optical techniques for wavelength conversion such as second-harmonic generation, which require high-intensity incident radiation, exciton fission and fusion operate in compact solid-state devices under incoherent and low-intensity illumination^7–9^.

Exciton fission and fusion both couple one singlet (spin zero) exciton to two triplet (spin one) excitons. In fission, for example, the initial state is one singlet exciton. The final state is two, independent, triplet excitons, each with approximately half the energy of the initial state. Exciton fission and fusion processes are mediated by a TT exciton pair. The net spin of the TT pair may be singlet, triplet or quintet. The quintet TT pair is particularly notable because it has a total spin of 2. This is unusual in organic semiconductors, in part because quintet excitons are typically optically inaccessible from a singlet ground state, and quintet excitons have much higher energies than singlets or triplets. Quintet TT states, however, can be energetically accessible because of the much weaker exchange interactions in TT states. Consequently, quintet TT states are notably involved in the efficiency and dynamics of exciton fission and fusion. Despite their clear importance, the generation, dynamics and control of quintet states remain challenging. To date, studies of quintet TT states have required very large magnetic fields or low temperatures, which limits the practical impact of quintet TT state engineering in many applications.

**Fig. 1:**
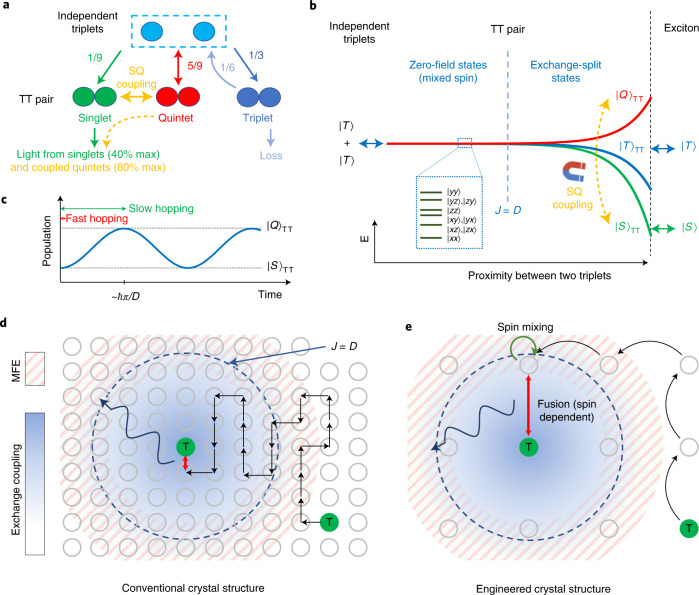
Spin dynamics of the triplet fusion process and a strategy for enabling singlet–quintet (SQ) coupling. **a**, Schematic illustration of TT pair dynamics. More than half of the TT pairs form quintet states initially, thus control of SQ coupling can substantially impact triplet fission and fusion efficiencies. **b**, Energy levels and spin states of nine eigenstates of TT pairs as a function of the distance between two triplet excitons. As shown in the inset, in a separated TT pair, the eigenstates are mixed SQ or triplet–quintet states governed by the zero-field splitting defined by the parameters *D* and *E* (ref. ^50^). The intertriplet exchange interaction, *J*, dominates as the triplets get closer, eventually yielding pure-spin eigenstates. **c**, A resonant magnetic field can drive an oscillation between the singlet and quintet states. For SQ splitting on the order of *D*, the spin transition time is ∼*ℏ*π*/D*. **d**,**e**, Spatial description of triplet fusion in molecular solids (**d**) and MOFs (**e**). Moderate applied magnetic fields *μB* ≈ *D* reshuffle the singlet, triplet and quintet character of TT pairs in the shaded magnetic field effect (MFE) regions, generating both the conventional Merrifield MFE and potentially SQ resonances. TT states with weaker exchange splitting *J* ≈ *D* may be precursors to exciton formation in MOFs. In a typical densely packed molecular solid, however, triplets migrate rapidly relative to *ℏ*π*/D* and fusion typically occurs from a triplet pair with exchange splitting *J* ≫ *D*. This means that, in molecular solids, SQ resonances may only be observed under very high applied magnetic fields *μ*_Β_*B* ≫ *D* and low temperature, if at all.

In the late 1960s, Merrifield verified the typical mechanism of exciton fission and fusion by studying these processes in the presence of a magnetic field^10–12^. In the Merrifield model, the singlet character of the TT pair determines its coupling to the singlet exciton. There are nine eigenstates for the TT pair, and as shown in Fig. 1a, the overall fractional character of the singlet, triplet and quintet states is 1/9, 3/9 and 5/9, respectively. If the quintet exciton is energetically inaccessible, then the quintet TT states cannot form a singlet exciton, dissociating instead into independent triplet excitons. Of the remaining TT states, 25% can yield an emissive singlet exciton. The triplet TT states can annihilate one of the triplets, yielding a maximum fusion efficiency of 40%^13^. It is notable, however, that quintets possess the same exchange symmetry as singlet TT states, meaning that under the appropriate magnetic field, a resonance can be engineered between singlet and quintet TT states. If we exploit the common exchange symmetry of singlet and quintet TT states, and couple them together, then potentially two-thirds of TT states can yield an emissive singlet exciton. The remaining TT states again annihilate one of the triplets, increasing the maximum fusion efficiency to 80%. Thus, quintet state engineering is capable of doubling the efficiency of optical upconversion. More broadly, the singlet–quintet (SQ) resonance is expected to be similarly useful for applications in quantum information that rely on the quintet.

In this work, we introduce a route to control quintet state dynamics by crystal structure engineering. We propose that a porous, ordered structure is particularly advantageous for SQ mixing because of weak intertriplet exchange coupling and slow excitonic hopping. Indeed, on the basis of the magnetic field effect (MFE) of triplet-fusion-based upconversion emission, we report here the observation of a distinct SQ resonance at room temperature under a low magnetic field of just 0.14 T.

## A strategy for quintet state engineering

Of the many possible TT states with varying triplet separation, the focus for achieving higher fusion efficiencies is the TT pair that couples directly to the singlet exciton. The aim is to exploit the energetic inaccessibility of the quintet exciton, and ensure that TT pairs in the quintet configuration form a singlet exciton rather than dissociating back into two independent triplets. Two design rules are crucial to achieve this. First, singlet and quintet states should be resonant in the immediate TT precursor to exciton fusion. If this condition is met, all quintet states can be directly harvested to singlet excitons if there is sufficient SQ mixing. Second, the precursor state should exhibit a sufficiently long lifetime to allow SQ mixing. If the TT pair quickly dissociates back to independent triplets, then SQ mixing will be ineffective.

Conventional molecular solids do not typically fulfil these two crucial design rules. As shown in Fig. 1b, intertriplet exchange interactions in conventional materials are strong relative to typical zero-field splitting because of the high density of triplet sites and the consequent small separation between the triplets in the precursor TT state. Indeed, a typical molecular zero-field splitting parameter, *D*, for triplet excitons is *D* ≈ 10 μeV (refs.^14,15^), whereas the intertriplet exchange interaction can be on the order of *J* ≈ 10 meV between nearest neighbours^16^. Unless the applied magnetic field is strong (*μ*_Β_*B* ≈ *J*), it will not overcome the intertriplet exchange interactions in closely spaced TT pairs, breaking the first design rule. Further, even if the singlet and quintet TT states are coupled by applying a strong magnetic field, the spin mixing is slow relative to exciton hopping or fluctuation of the exchange interaction (Fig. 1c), breaking the second rule. For SQ splitting ∼*D*, the transition time *ℏ*π*/D* ≈ 200 ps, which is slower than typical hopping rates in organic semiconductors^17,18^.

The Merrifield model typically used to describe fusion and fission MFEs in molecular solids does not consider exchange splitting. Indeed, the Merrifield MFE observed under moderate fields (∼0.1 T) is generated within widely spaced TT pairs with *J* ≈ 0 that can rapidly collapse to an exciton while preserving their total spin. Conventional molecular materials under high magnetic fields (>2 T), however, exhibit non-Merrifield behaviour, including sharp resonances in the MFE of singlet fission^19–22^. In these studies, the applied magnetic field overcomes the exchange splitting in more closely spaced TT pairs, thereby enabling mixing between singlet and quintet TT states. The observed effects are more pronounced at low temperatures, consistent with the additional expectation that triplet hopping should also be retarded for effective spin mixing. To date, only very weak exchange features have been observed at room temperature, and solely under a high magnetic field, that is, above 2 T.

To achieve effective SQ coupling in TT states at room temperature under a moderate magnetic field (<0.2 T), we seek to exploit the unique structural properties of metal–organic frameworks (MOFs). These are microporous crystalline materials based on organic and inorganic building blocks^23–25^. The porous structure of MOFs enables the incorporation of spatially separated triplet sensitizing molecules that become part of a highly ordered crystal structure. Because there is an enormous library of possible linkers, MOFs offer a vast compositional and structural platform for studying triplet-fusion-based upconversion processes. Such processes have indeed been demonstrated in several MOFs^26–30^, with a particular focus on upconversion performance and applications.

Most relevantly, evidence of upconversion from MOFs with large unit cells^26–30^ implies that MOFs may support triplet fusion from a distanced TT pair where the exchange interaction is comparable to the zero-field splitting (Fig. 1e). The MOF structure can also be used to engineer the triplet hopping rate, which competes with triplet fusion by separating the TT state. Finally, the MOF structure can suppress fluctuations in the exchange coupling during SQ mixing. The intertriplet exchange interaction is determined by the orbital overlap between the triplets, such that it can be dynamically modulated by molecular vibrations^31^ or exciton hopping. Indeed, whereas molecular solids are held together by weak Van der Waals interactions that give rise to various low-frequency phonons^32^, linkers in MOFs are rigidified by stronger coordination bonds to the metal centres. Simulations show that MOFs reduce low-frequency phonons^33^ and torsional vibrations^34^. These vibronic and hopping restrictions enable stronger coupling between singlet and quintet TT states and minimize efficiency losses due to the competing process of separating TT states back to independent triplets.

## Intertriplet exchange interaction and triplet fusion MFE

To elucidate the role of the exchange coupling in triplet fusion, we calculate the MFE on a triplet pair’s nine eigenstates with different exchange coupling and corresponding triplet fusion rates, in analogy with the calculations for singlet fission^22^. The Merrifield model was used with the spin Hamiltonian of equation (1).

1 $$\begin{array}{l}{{{\boldsymbol{H}}}} = g\mu _{\mathrm{B}}{{{\boldsymbol{B}}}} \cdot \left( {{{{\boldsymbol{S}}}}_1 + {{{\boldsymbol{S}}}}_2} \right) + D\left( {{{{\boldsymbol{S}}}}_{z1}^2 + {{{\boldsymbol{S}}}}_{z2}^2} \right)\\\qquad + E\left( {{{{\boldsymbol{S}}}}_{x1}^2 + {{{\boldsymbol{S}}}}_{x2}^2 - {{{\boldsymbol{S}}}}_{y1}^2 - {{{\boldsymbol{S}}}}_{y2}^2} \right) - 2J\,{{{\boldsymbol{S}}}}_1 \cdot {{{\boldsymbol{S}}}}_2\end{array}$$

The first term is the Zeeman interaction where *g, μ*_B_*,* ***B*** and ***S*** are the *g*-factor, Bohr magneton, magnetic field and spin operators, respectively. The second and third terms are the intratriplet dipole–dipole coupling, and *D* and *E* are zero-field splitting parameters. The fourth term is the intertriplet exchange interaction where *J* is the exchange constant. Other weak spin interactions, such as hyperfine coupling, are ignored^12^. A detailed procedure is described in Supplementary Note 1.

**Fig. 2:**
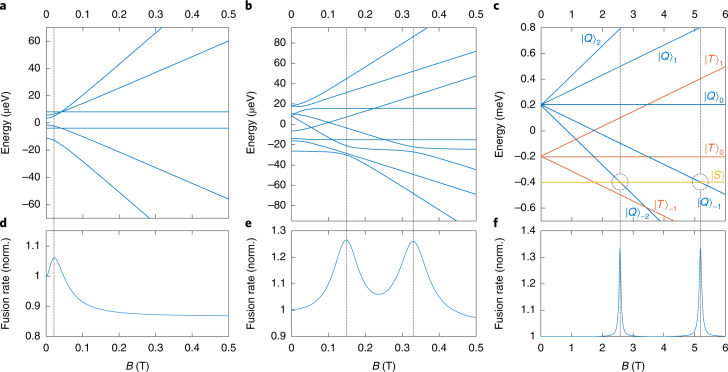
Magnetic field effect (MFE) on triplet–triplet pair eigenstate energies and triplet fusion rate for three different values of the intertriplet exchange coupling. **a**–**c**, MFE on the eigenstates of TT pairs with different intertriplet exchange interactions: *J* = 0 μeV (**a**), *J* = –6.1 μeV (**b**) and *J* = –100 μeV (**c**). **d**–**f**, MFE on the normalized triplet fusion rates that are proportional to singlet characteristics of TT pairs for *J* = 0 μeV (**d**), *J* = –6.1 μeV (**e**) and *J* = –100 μeV (**f**). When there is no exchange interaction (**a**,**d**), the singlet-like TT pair population is modulated by the intratriplet dipole–dipole interaction and Zeeman interaction. These are Merrifield-type MFE characteristics, as the model assumes *J* = 0. When a strong exchange interaction is present (**c**,**f**), each eigenstate represents the total spin states of TT pairs. The fusion rate increases when quintet states mix with singlet states near the avoided level crossings. When the exchange coupling is comparable to intratriplet dipole coupling (**b**,**e**), each state is not a pure spin state, but contains a dominant spin character. The fusion rate increases near the avoided crossing of singlet-dominant and quintet-dominant states. The resonance positions deviate from their expected doubling of the field strength, as modulated by the relative sign of *D* and *J*. Note that these calculations assume a single crystal with zero-field splitting parameter *D* = 8.5 μeV, whereas the experimental MFE curve was obtained from polycrystalline materials. Note also that *J* is a function of TT separation, as described in Fig. 1.

The exchange interaction substantially affects the MFE shape, as shown in Fig. 2. When there is no applied field and no exchange coupling in the TT state (Fig. 2a,d), the intratriplet dipole coupling generates three spin states that contain singlet characteristics. Weak Zeeman interactions mix the singlet character into more states, thereby increasing the fusion rate. However, only two states contain singlet characteristics under strong Zeeman interactions, slowing the fusion rate under stronger applied magnetic fields^12^.

When the exchange coupling is much stronger than the zero-field splitting, eigenstates are pure spin states (Fig. 2c,f). Zeeman interactions do not change the distribution of singlet character unless a strong resonant magnetic field is applied to match the SQ splitting. As shown in Fig. 2f, under resonant conditions, singlet and quintet states can be mixed near the avoided level crossings, resulting in a faster fusion rate to the singlet exciton. The second resonance occurs at a magnetic field strength double that of the first resonance, which helps identify exchange coupling. Also, the level-crossing features appear at a high magnetic field because of the strong exchange interaction. Note that the mixing between singlet and triplet states is symmetry-forbidden unless the system has a symmetry-breaking factor.

In a MOF, the exchange interaction can be much weaker because of the large spatial separation between the organic linkers. When the exchange coupling is comparable to the intratriplet dipole interaction (Fig. 2b,e), eigenstates are not pure spin states because of the zero-field splitting, but they contain a large portion of a spin character due to the exchange interaction. Unlike the case of negligible exchange splitting, the initial rate increase with applied magnetic field is not due to a combination of the zero-field splitting and weak Zeeman interaction. Instead, there are two peaks from the spin mixing between a singlet-dominant TT state and quintet-dominant TT states. Note that the rate increase occurs at a low magnetic field. Also, the two resonances do not occur with the expected doubling of magnetic field strength. Depending on the relative sign and magnitude of *D* and *J*, the resonances may be observed closer or further apart. This unique MFE, distinct from the Merrifield curve, can confirm the spin mixing from the exchange interaction.

## Triplet-fusion-based upconversion in NU-1000

**Fig. 3:**
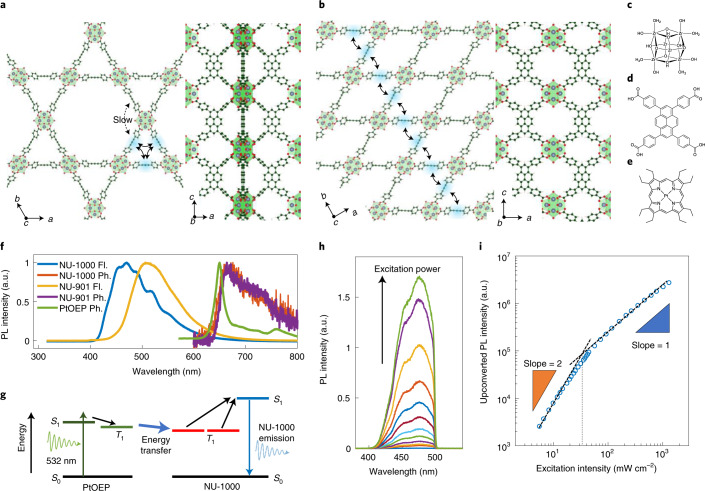
Portions of the structures of NU-1000 and NU-901, a triplet sensitizer, and their photophysical properties. **a**–**e**, Structure of NU-1000 (**a**), NU-901 (**b**), oxo-Zr_6_ SBU (**c**), H_4_TBAPy (linker) (**d**) and PtOEP (triplet sensitizer) (**e**). Note the unique placements of the ligand molecules in the MOFs that are distinct from molecular solids. PtOEP molecules are incorporated into the large pores of the MOFs. **f**, Photoluminescence spectra of NU-1000, NU-901 and PtOEP. Blue and red curves represent NU-1000 fluorescence (Fl.) and phosphorescence (Ph.), respectively. Yellow and purple curves show NU-901 fluorescence and phosphorescence spectra, respectively. The PtOEP phosphorescence (green curve) is higher in energy than those of either MOF, ensuring efficient Dexter energy transfer from PtOEP to the MOFs. **g**, Schematic of the triplet-fusion-based photon upconversion process in the NU-1000:PtOEP system. **h**, The upconverted emission spectrum of NU-1000:PtOEP excited with a *λ* = 532 nm laser. **i**, Pump-power dependence of upconverted emission exhibiting a slope change from 2 to 1. Such a power dependence transition is observed when the dominant decay mechanism for triplet excitons shifts from a first-order process to TT fusion, and coincides with maximizing the upconversion efficiency. The upconversion threshold intensity is 35 mW cm^−^^2^.

To verify our proposed model for triplet fusion in MOFs, we have selected two pyrene-based materials, NU-1000 and NU-901. As shown in Fig. 3, the two materials are made from the same organic ligand, tetrakis(*p*-benzoic-acid)pyrene (H_4_TBAPy), and secondary building units (SBUs) of similar metallic composition. However, the two MOFs have different crystal structures: NU-1000 is mesoporous and bears **csq** topology^35^, whereas NU-901 is microporous with **scu** topology^36^. Notably, the excitons in NU-1000 can be confined around the small triangular pore, while excitons in NU-901 can travel in the *a*–*b* plane, as shown in Fig. 3a,b. Naturally then, NU-1000 and NU-901 differ in their triplet hopping rates, allowing us to interrogate the effect of the lifetime of the TT state on the efficiency of SQ coupling.

NU-1000 is an archetypal MOF with excellent stability^35^ and various attractive properties^37–41^. Its organic linker is attractive for our purposes because it has a pyrene core. The triplet level in pyrene is close to half of its singlet; photon upconversion has been demonstrated with pyrene and its derivatives^42,43^. To determine the triplet energy level of NU-1000 and NU-901, we measured their phosphorescence spectra at 80 K. As shown in Fig. 3f, the phosphorescence emission peak of NU-1000 is 1.9 eV, substantially lower than the singlet emission at 3.0 eV. In contrast, NU-901 has a singlet level of 2.5 eV and a triplet of 1.9 eV. To generate triplet excitons in the two MOFs, we used platinum octaethylporphyrin (PtOEP) as a triplet sensitizer. Its triplet level of 2.0 eV is higher than that of the MOFs, ensuring an efficient Dexter energy transfer from PtOEP to the MOFs. The schematic illustration of the upconversion process is summarized in Fig. 3g. Due to the large pores of the MOFs, PtOEP molecules can be inserted into the MOFs without destroying the overall structure (see Methods for sample preparations).

NU-1000:PtOEP crystals show upconverted blue emission when excited with a 532 nm green laser (Fig. 3h). The excitation power dependence of the upconversion shows a quadratic-to-linear transition, a signature of TT annihilation-based upconversion (Fig. 3i). The transition intensity is 35 mW cm^−^^2^. As a benchmark, total solar irradiance is 100 mW cm^−^^2^ under AM1.5 conditions and the partial solar irradiance for a ∆*λ* = 20 nm window centred at *λ* = 530 nm is 3 mW cm^−^^2^. The photoluminescent quantum yield (PLQY) *ϕ*_PL_, external quantum efficiency EQE, and the efficiency of singlet-state generation from absorbed photon pairs, $\phi _{\mathrm{UC,s}}^\prime$, normalized to the PLQY of the MOFs, is *ϕ*_PL_ = 2.1% ± 0.1% and 0.26% ± 0.09%, EQE in the range of 2.5 × 10^−3^% to 3.7 × 10^−3^% and 1.0 × 10^−4^% to 3.1 × 10^−4^%, and $\phi _{\mathrm{UC,s}}^\prime$ in the range of 0.54–1.8% and 0.14–0.45% for NU-1000 and NU-901, respectively (Methods and Supplementary Note 2). These efficiencies are among the highest demonstrated by MOF-based upconversion in a solid-state system^44^, and compare to $\phi _{\mathrm{UC,s}}^\prime=$ 2.46% in a highly optimized green–blue multilayer structure based on small-molecular-weight thin films^45^, $\phi _{\mathrm{UC,s}}^\prime=$ 12.7% in a polymer-based system sensitized by PtOEP^46^, and $\phi _{\mathrm{UC,s}}^\prime=$ 1.6% in a quantum-dot-sensitized infrared-to-visible structure^47,48^.

## Singlet–quintet resonance in NU-1000

**Fig. 4:**
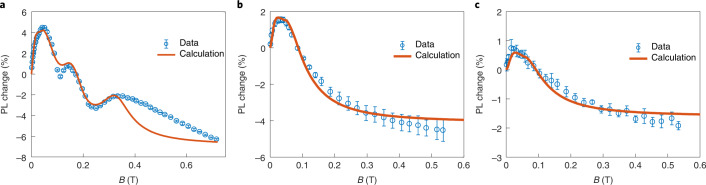
Magnetic field effects on upconverted emission. **a**, NU-1000:PtOEP has an unique MFE with two additional distinct peaks around 0.14 and 0.33 T. **b**,**c**, NU-901:PtOEP (**b**) and a physical mixture of H_4_TBAPy:PtOEP (10 wt%) (**c**) show the conventional MFE for triplet fusion, confirming the critical role of structure in NU-1000 for enabling anomalous MFE behaviour with NU-1000:PtOEP. Error bars are the standard deviation of the mean and are averaged from five, seven and four independent sweeps for **a**, **b** and **c**, respectively.

To investigate the detailed spin dynamics of triplet fusion in MOFs, we measured the upconverted emission intensity under an external magnetic field. Remarkably, NU-1000:PtOEP shows an anomalous MFE curve with two additional distinct peaks (Fig. 4a), which diverges from the conventional Merrifield-type behaviour of molecular solids. Highlighting the differences between the two MOFs, triplet fusion in NU-901 follows the Merrifield-type MFE (Fig. 4b), suggesting that the origin of the anomalous MFE observed for triplet fusion with NU-1000 lies in its unique structural features.

Two additional control experiments confirm the origin of the anomalous behaviour in NU-1000. First, we measure the MFE of upconversion for a thin film made from a physical mixture of H_4_TBAPy ligand and PtOEP (10 wt%). As shown in Fig. 4c, this mixture does show upconversion that nevertheless follows the regular curve shape conforming to the Merrifield model. Second, the use of a different sensitizer, palladium octaethylporphyrin (PdOEP), also leads to upconversion upon insertion in NU-1000, and the fusion MFE of NU-1000:PdOEP also generates the abnormal MFE with the same peak positions (Supplementary Fig. 2). Altogether, these data demonstrate that the anomalous behaviour of NU-1000 is independent of the sensitizer, but strictly arises because of the particular spatial arrangement and separation of TBAPy^4–^ ligands within NU-1000.

Importantly, the MFE curve of NU-1000 can be explained by the weak exchange interaction proposed in our model above. As shown in Fig. 1e, the porous MOF structure is expected to offer a weak exchange coupling that allows sufficient time for SQ mixing. Indeed, a reasonable fit for the MFE curve is obtained with the Johnson–Merrifield model^49^ using the spin Hamiltonian in equation (1), as shown in Fig. 4a. This fit is calculated by combining the MFE of non-exchange-coupled triplet pairs (Fig. 2d) and the weak exchange-coupled pairs (Fig. 2e). Three parameters determine the three peak positions of the anomalous curve, and the fit gives *D* = 8.50 μeV, *E* = −1.17 μeV and *J* = −6.1 μeV. Notably, the zero-field splitting parameters obtained by electron paramagnetic resonance match the resonance peak position (Supplementary Fig. 3). Other fitting parameters are *k*_*S*__,*J*=0_ = 1.0 × 10^8^ s^−1^, *k*_−1,*J*=0_ = 1.7 × 10^8^ s^−1^, *k*_*S*,*J*≠0_ = 5.0 × 10^9^ s^−1^ and *k*_−1,*J*≠0_ = 3.3 × 10^9^ s^−1^ where *k*_*S*_ and *k*_−1_ are the fusion and dissociation rates from a TT pair. For NU-901 (Fig. 4b) and the ligand (Fig. 4c) MFE calculations, similar zero splitting parameters were used with *J* *=* 0. The only major difference is *k*_**–**1_, which is 5.0 × 10^8^ s^−1^ for NU-901 and 1.0 × 10^9^ s^−1^ for the ligand. Details of the calculations are described in Supplementary Note 1.

The absence of resonant peaks in the MFE for fusion with NU-901 confirms that confined excitons are needed to ensure that spin mixing can compete effectively with triplet hopping and exciton formation. Excitons in NU-901 can migrate ad infinitum in the *a*–*b* plane via the first nearest-neighbour (NN) hopping (Fig. 3b), whereas excitons in NU-1000 are confined to triangular motifs of the first NN interactions, and require the second, much more distanced, NN interaction to travel further in the *a*–*b* plane (Fig. 3a). The hopping rate for this more distanced NN interaction is calculated by constrained density functional theory (CDFT) to be three orders of magnitude slower than the first NN hopping rate (Supplementary Note 3). Therefore, triplet excitons in NU-1000 are effectively localized, making unproductive triplet diffusion less competitive with SQ mixing. Indeed, Monte Carlo simulations of triplet-pair trajectories support an enhancement in the effectiveness of spin mixing for the triplet pair in NU-1000 relative to the TT state in NU-901 (Supplementary Note 3 and Supplementary Fig. 4).

The CDFT calculations of intertriplet exchange coupling support that the observed resonance peak originates from the first NN interactions in NU-1000. Whereas the first NN interaction might be of the same order of magnitude as the experimental value, the second NN interaction is much weaker. For example, the PBE0 functional predicts the NN and the second NN exchange couplings to be 18 μeV and 0.017 μeV, respectively (Supplementary Note 4). Because the first NN interactions are the immediate precursor to fusion, quintet TT states can be mixed effectively with singlet TT states and be harnessed for optical upconversion.

The resonance peak observed from NU-1000 at the low magnetic field of only 0.14 T provides experimental proof of a route that utilizes quintet states in optical upconversion. Although the experimentally absorbed photoluminescence (PL) increase at the resonance is only approximately 2% when accounting for the background Merrifield MFE, further optimization of the MOF structure is likely to improve the efficiency by engineering key parameters such as the fusion rate, hopping rate, decoherence time and the zero-field splitting. Indeed, as discussed in Supplementary Note 5 and Supplementary Fig. 5, the efficiency can potentially increase by 65% when the kinetic parameters are tuned.

In conclusion, the SQ TT state resonances in NU-1000 demonstrate that, in contrast to their molecular and polymeric counterparts, MOFs enable precise engineering of interexciton exchange coupling and spin dynamics. MOFs thus offer particular promise for controlled access and coupling to quintet states, which have been rarely studied despite their importance in multiexciton processes such as exciton fission and fusion. Not least, these results demonstrate that the unconventional structure and immense structural and compositional tunability of MOFs provide a compelling platform for elucidating detailed mechanisms of multiexcitonic processes.

## Methods

### Materials

All reagents were purchased commercially and used without further purification. PtOEP and PdOEP were purchased from Lumtec. H_4_TBAPy was synthesized following literature^51^ or purchased from Sigma-Aldrich.

### Synthesis of NU-1000

The synthetic procedure was adapted from a reported literature method, which involves using trifluoroacetic acid (TFA) as a modulator for ensuring phase purity^52^. ZrCl_4_ (70 mg, 0.30 mmol) and benzoic acid (2 g, 16.38 mmol) were mixed in 6 ml diethlyformamide (DEF) and sonicated until a clear solution was obtained. The resulting solution was heated in an oven at 100 °C for 1 h, allowing for the formation of zirconium nodes. H_4_TBAPy (40 mg, 0.06 mmol) was suspended in 4 ml DEF and heated in an oven at 100 °C for 1 h. After cooling down to room temperature, H_4_TBAPy solution and TFA (40 μl, 0.52 mmol) were added to the zirconium-node-containing solution. The resulting mixture was sonicated for 10 min, followed by heating in an oven at 120 °C for 24 h. Upon gradual cooling to room temperature (oven, ∼8 h), light-yellow polycrystalline NU-1000–TFA was obtained, which was collected by centrifugation (4 min, 3,500 r.p.m., 1,972 × *g*), and washed with dimethlyformamide (DMF) (three times, 10 ml each, soaked ∼1 h between washes). The coordinated TFA was removed by suspending NU-1000–TFA crystals in 13 ml DMF, and 8 M aqueous HCl (0.5 ml) was added. The mixture was heated in an oven at 100 °C for 18 h. After gradual cooling to room temperature, the polycrystalline powder of NU-1000 was isolated by centrifugation, washed with DMF (three times, 10 ml each), and then with acetone (three times, 10 ml each) (soaked ∼1 h between all washes). The resulting material was dried in a vacuum oven at 80 °C for 1 h to yield light-yellow NU-1000 crystals.

### Synthesis of NU-901

The synthetic procedure was slightly modified from a literature report^53^. Zr(acac)_4_ (97 mg, 0.2 mmol) and 4-aminobenzoic acid (1.51 g, 11 mmol) were mixed in 4 ml of DMF in a 20 ml vial and ultrasonicated for 30 min. The vial was heated in an oven at 80 °C for 1 h. Then, H_4_TBAPy (40 mg, 0.06 mmol) was added and sonicated for 10 min. The vial was incubated in a preheated oven at 100 °C for 18 h, resulting in yellow MOF crystals. The MOFs were isolated by centrifuge (5 min, 4,000 r.p.m., 2,576 × *g*) and solvent exchanged with fresh DMF four times (∼15 ml each) followed by methanol three times (∼15 ml). To activate, the MOFs were suspended in 12 ml DMF, and 0.5 ml of 8 M aqueous HCl was added. The vial was heated in an oven at 100 °C for 18 h. The powder was washed with DMF and acetone three times each. It was activated in a vacuum oven at 80 °C for 1 h prior to PtOEP incorporation.

### Incorporation of guest molecules into MOFs and sample preparation for optical measurements

First, 4 mg of PtOEP (or PdOEP) was dissolved in 1 ml of toluene in a nitrogen glovebox. NU-1000 or NU-901 were soaked in the solution for 1 week. The solution was stirred to accelerate the incorporation. Then, 4 ml of toluene was added to the solution, and the solution containing NU-1000:PtOEP crystals was dropcasted on a 1 inch quartz substrate. The crystals were gently rinsed with toluene and dried. The sample was encapsulated with an ultraviolet-curing epoxy (OG159-2, Epoxy Technology) in a nitrogen glovebox (O_2_ < 0.1 p.p.m., H_2_O < 0.1 p.p.m.). We also tested a reduced incorporation time. NU-1000 was soaked in the solution for 30 min at 60 °C. The crystals were not rinsed with toluene after the dropcasting. The anomalous magnetic field effect was observed for both conditions.

### PL measurement

The PL spectrum was measured with a spectrometer (SP2300 and PIXIS 100, Princeton Instruments). The laser intensity was determined by a powermeter (PM100A, Thorlabs) and a CMOS camera (DCC1545M, Thorlabs). The NU-1000 phosphorescence spectrum was collected at 80 K using a liquid-nitrogen-cooled cryostat. Two phase-locked chopper wheels (MC2000B, Thorlabs) were used to collect a delayed portion of the photoluminescence.

### PLQY and upconversion efficiency measurement

The PLQY of the MOF-only samples, and upconversion efficiencies of PtOEP-incorporated samples were measured in an integrating sphere (RTC-060-SF, Labsphere) following the method developed by de Mello et al.^54^. As specified in this method, three measurement configurations were carried out: (A) sample out of the sphere, (B) sample in the sphere but not directly excited by the laser, and (C) sample in the sphere and directly excited by the laser. The first-pass absorption (Abs) was obtained via:

$${\mathrm{Abs}} = 1 - \frac{{L_{\mathrm{C}}}}{{L_{\mathrm{B}}}},$$

where *L*_A_*, L*_B_ and *L*_C_ are the measured laser powers normalized by pump photon energy in configurations A, B and C as described above. The PLQY can be determined by:

$${\mathrm{\phi}_\mathrm{PL}} = \frac{{P_{\mathrm{C}} - \left( {1 - {\mathrm{Abs}}} \right) \times P_{\mathrm{B}}}}{{L_{\mathrm{A}} \times {\mathrm{Abs}}}},$$

where *P*_A_*, P*_B_ and *P*_C_ are the measured emission powers normalized by emissive photon energy in configurations A, B and C.

A 405 nm laser (maximum power, 4.5 mW; CPS405, Thorlabs) was used to measure the PLQYs of the MOFs, while upconversion efficiencies were measured by a 532 nm laser (maximum power, 4.5 mW; CPS532, Thorlabs) excitation source. The laser beam was focused down to a spot size similar to that of PL measurement. Emission from the output of the sphere was redirected and collimated by a 90° off-axis parabolic mirror (MPD00M9-F01) before being focused onto a spectrometer (SP2300 and PIXIS 100, Princeton Instruments). Such a redirected output setup allowed application of a shortpass filter (FESH0500, Thorlabs) to exclude laser signal when capturing upconverted PL, making it possible to increase the integration time and slit size of the spectrometer to improve the signal-to-noise ratio of upconverted PL. The upconversion efficiency (*ϕ*_UC_), defined as the fraction of the number of upconverted photons to the number of absorbed photons, can be obtained by:

$$\phi _{\mathrm{UC}} = 2 \times {\mathrm{EQE}}/{\mathrm{Abs}},$$

where EQE is the ratio of emitted upconverted photons to incident pump photons. Accounting for reabsorption in an integrating sphere, *ϕ*_UC_ was determined similarly to PLQY, that is:

$$\phi _{\mathrm{UC}} = 2 \times \frac{{P_{{\mathrm{C}}, {\rm{UC}}}\, - \, \left( {1 - {\mathrm{Abs}}_{{532}\, {\rm{nm}}}} \right)\, \times \, P_{{\mathrm{B}}, {\rm{UC}}}}}{{L_{{\mathrm{A}}, 532 \, {\rm{nm}}}\, \times \, {\mathrm{Abs}}_{{532 \,{\rm{nm}}}}}}$$

Finally, the upconverted singlet-state yield (*ϕ*_UC,s_), defined as the number ratio of upconverted singlet excitons to absorbed photon pairs, was determined by:

$$\phi _{{\mathrm{UC}},{\rm{s}}} = \phi _{\mathrm{UC}}/{\mathrm{PLQY}}_{{405\, {\rm{nm}}}}$$

### Magnetic field effect measurement

A 532 nm laser (maximum power, 4.5 mW; CPS532, Thorlabs) was used as the excitation source, and the laser beam was chopped and filtered with a 532 nm bandpass filter. A silicon detector (818-UV, Newport) measured upconverted emission intensity together with a lock-in amplifier (SR830, Stanford Research Systems). A 500 nm shortpass filter and a 532 nm notchpass filter were placed in front of the detector to collect only the upconverted emission. An electromagnet was switched between positive and zero magnetic fields every 20 s, and the PL change was estimated from four cycles. The magnetic field was recorded with a gaussmeter (HMMT-6J04-VF, Lakeshore). The four cycles of measurement were repeated for different magnetic fields to obtain Fig. 4a. The measurement was swept from a high field to a low field, and the data in Fig. 4a are an average of five independent sweeps. For NU-901:PtOEP, the PL change was estimated from three cycles, and Fig. 4b is an average of seven independent sweeps. The upconversion emission of ligand:PtOEP is weak, so an intense laser (4 W; Verdi G18, Coherent) was used. To avoid the photodegradation of the ligand, the PL change was estimated from two cycles and Fig. 4c is an average of four independent sweeps.

### Electron paramagnetic resonance measurement

Electron paramagnetic resonance (EPR) spectroscopy was performed on the MOF powders packed in 4-mm-outside-diameter thin-wall quartz EPR sample tubes (Wilmad) on a Bruker EMX-Plus spectrometer equipped with ER4119HS high-sensitivity X-band resonator at 9.37 GHz. The sample tube was encapsulated in a nitrogen glovebox with an ultraviolet-curing epoxy (OG159-2, Epoxy Technology). Cryogenic temperature was achieved with a Bruker/ColdEdge 4 K waveguide cryogen-free cryostat. Photoexcitation of the sample was performed by illuminating the sample with a 365 nm light-emitting diode through the oval windows on the resonator. Simulation of the EPR spectrum was performed with the EasySpin^55^ package in Matlab.

### Energy-dispersive spectroscopy

Ex situ energy-dispersive spectroscopy elemental mapping was collected at the MIT MRSEC (formerly the Center for Materials Science and Engineering, or CMSE) on a JEOL 2010 FEG analytical electron microscope equipped with an Oxford Instruments ULTIM MAX detector at an operating voltage of 10 kV. The measurements were conducted while the specimens were under a high vacuum. The specimens were prepared by dropcasting the NU-1000/PtOEP or NU-901/PtOEP toluene suspension onto a silicon wafer. The dropcast samples were gently washed with toluene until no visible red colour of excess PtOEP came off in the rinses. For every sample, elemental mapping was carried out two or three times on different randomly chosen areas.

## Online content

Any methods, additional references, Nature Research reporting summaries, source data, extended data, supplementary information, acknowledgements, peer review information; details of author contributions and competing interests; and statements of data and code availability are available at 10.1038/s41563-022-01368-1.

## Supplementary information

Supplementary Information Supplementary Notes 1–5 and Figs. 1–9.

## Data Availability

All data generated or analysed during this study are included in this published article (and its Supplementary Information files).

## Code availability

The computational codes used to calculate the spin Hamiltonian and Monte Carlo models are available from the corresponding authors upon reasonable request. The ab initio calculations of energies and couplings were performed using Q-Chem (https://www.q-chem.com/), with a modification to increase the number of digits that are printed. The results are provided in this paper and its Supplementary Information.
