# Supplementary material for: Exchange controlled triplet fusion in metal–organic frameworks
Source: Nat Mater. 2022 Oct 6;21(11):1275–81. doi: 10.1038/s41563-022-01368-1 (PMC9622415; doi:10.1038/s41563-022-01368-1)
Supplement: Supplementary file 1 — Supplementary Notes 1–5 and Figs. 1–9. [file 41563_2022_1368_MOESM1_ESM.pdf]

nature materials

Article https://doi.org/10.1038/s41563-022-01368-1

Exchange controlled triplet fusion in metal–organic frameworks

In the format provided by the authors and unedited

Supplementary materials for

Exchange controlled triplet fusion in metal-organic frameworks

Dong-Gwang Ha[1], Ruomeng Wan[1], Changhae Andrew Kim[1], Ting-An Lin[2], Luming Yang[1], Troy Van Voohris[1], Marc A. Baldo[2], and Mircea Dincă[1]

[1] Department of Chemistry, Massachusetts Institute of Technology, Cambridge, MA 02139, USA

[2] Department of Electrical Engineering and Computer Science, Massachusetts Institute of Technology, Cambridge, MA 02139, USA

This file includes:

Supplementary Notes 1 to 5

Figs. S1 to S9

Supplementary Note 1. The MFE calculation of triplet fusion

The fusion process can be kinetically described as

$$\mathrm{T + T} \underset{k_{-1}}{\overset{k_1}{\rightleftharpoons}} \mathrm{TT} \xrightarrow{k_\mathrm{S}} \mathrm{S}$$

where the fusion rate (k_s) is governed by a spin character of the triplet pair state (TT). The plots in Fig. 2 are calculated by the simple Merrifield model[1] to show the effect of the intertriplet exchange coupling. The parameters used in the calculations are k_{-1}/k_s= 1, D= 8.5 μeV, and E= –1.1 μeV, based on a combination of fits to EPR and MFE data.

The simple Merrifield model assumes stationary states of the spin Hamiltonian, only valid for a pair with long lifetime. We used the Johnson-Merrifield model that employs the full density matrix of the triplet pair spin states to better fit the experimental results[2]. The time dependence of the density matrix is,

$$\frac{\partial \rho_{mn}}{\partial t} = -\frac{i}{\hbar}[H,\rho]_{mn} - k_{-1}\rho_{mn} - \frac{1}{2}k_S\left(\delta_{m,0} + \delta_{n,0}\right)\rho_{mn} + k_1[T]^2 I \tag{1}$$

The first term is the time evolution of the density matrix by spin Hamiltonian. The second is the dissociation of a triplet pair, the third is the fusion from a triplet pair to a singlet exciton, and the last term is the triplet pair formation. The density matrix was solved numerically with the steady-state condition ($\frac{\partial \rho}{\partial t} = 0$) to calculate the fusion rate. The full elements of the spin Hamiltonian can be found in Ref. [3]. We have generated 1000 random molecular orientations and averaged the results to account for the polycrystalline sample.

To fit the data in Fig. 4a, we combined two MFE curves (with/without the exchange interaction) at a 16: 84 ratio. For non-exchange coupled MFE, k_S= 1×10^8 s^{-1} , k_{-1}= 1.7×10^8 s^{-1} , J= 0 , D= 8.5 μeV and E= –1.17 μeV were used. For exchange-coupled MFE, k_S= 5×10^9 s^{-1} , k_{-1}= 3.3×10^9 s^{-1} , J= –6.1 μeV, D= 8.5 μeV and E= –1.17 μeV. Faster k_S and k_{-1} were used for the exchange-coupled pair, because an excitonic hopping changes the exchange interaction that reduces the TT pair lifetime under a specific exchange coupling. In contrast, many sufficiently separated triplet pairs have negligible J, so the hopping in non-exchange coupled regime does not affect the TT pair lifetime. Repeated experiments at different locations on a sample, and samples prepared under different conditions yielded MFE curves with the same peak positions, but the overall signal can shift as shown in Fig. S6. The peak positions are determined by J, D, and E. The various rates described above affect the intensities of the peaks. We propose that one possible explanation for the varying offset in the experimental data is different degrees of triplet-charge annihilation, and a monotonic Lorentzian curve[4] was added to simulate triplet-charge annihilation and correct the shift up to 2.5%.

For NU-901 (Fig. 4b) MFE calculations, the fitting parameters are k_S= 1.0×10^8 s^{-1} , k_{-1}= 5.0×10^8 s^{-1} , J= 0 , D= 9.0 μeV and E= –1.3 μeV. For the the ligand (Fig. 4c), k_S= 1.0×10^8 s^{-1} , k_{-1}= 1.5×10^9 s^{-1} , J= 0 , D= 10 μeV and E= –1.7 μeV.

Supplementary Note 2. Efficiency of upconversion

For excitonic upconversion systems, the upconversion efficiency *EQE* is defined as: [5]

$$EQE = \frac{photons\ out}{photons\ in} = \phi_{Abs} \times \phi_{ISC} \times \phi_{DET} \times \phi_{TTA} \times (1 - \phi_{BT}) \times \phi_{PL},$$

where ϕ_{Abs} is the fraction of incident photons absorbed, ϕ_{ISC} is the intersystem crossing efficiency of the sensitizer, ϕ_{DET} is the triplet transfer efficiency from the sensitizer to the annihilator, ϕ_{TTA} is the TTA efficiency in the annihilator, ϕ_{BT} is the singlet back transfer from the annihilator to the sensitizer, PtOEP, and ϕ_{PL} is the photoluminescence quantum yield (PLQY) of the annihilator.

The thickness of each MOF sample was observed to be highly spatially non-uniform, contributing to variation in the measured optical absorption, PLQY, and EQE.

Defining the maximum efficiency as 100%, the measurement of the internal quantum yield of upconversion, ϕ'_{UC}, as described in Methods, yielded (3.72×10^{-2})% and (1.18×10^{-3})% for NU-1000: PtOEP and NU-901: PtOEP, respectively.

To compare the spin-dependence of upconversion in MOFs to other solid-state excitonic systems, the net upconverted singlet state yield ($\phi'_{UC,s}$) can be normalized by the PLQY: [5]

$$\phi'_{UC,s} = 2 \times \phi_{ISC} \times \phi_{DET} \times \phi_{TTA} \times (1 - \phi_{BT}) = \frac{\phi'_{UC}}{\phi_{PL}}$$

The ϕ_{PL}'s of NU-1000 and NU-901 are measured (see Methods) to be (2.06±0.12)% and (0.26±0.09)%, respectively, with the error determined from measurements at three different locations on the sample. The range of measured $\phi'_{UC,s}$ for NU-1000 and NU-901 was (0.54% - 1.8%) and (0.14%-0.45%), respectively. Noting that the efficiency scales inversely with absorption, we assumed that the peak measured $\phi'_{UC,s}$ was the most accurate quantification of the internal processes given the presence of reabsorption losses.

	NU-1000					NU-901	
EQE (%)	0.0025	0.0020	0.0031	0.0028	0.0037	3.15×10^{-4}	1.03×10^{-4}
ϕ_{Abs} (%)	33.8	25.1	25.0	49.8	19.9	54.6	54.7
ϕ_{UC} (%)	0.0144	0.0158	0.0252	0.0112	0.0372	1.18×10^{-3}	3.60×10^{-4}
ϕ_{PL} (%)	(2.06±0.12)	(2.06±0.12)	(2.06±0.12)	(2.06±0.12)	(2.06±0.12)	(0.26±0.09)	(0.26±0.09)
$\phi'_{UC,s}$ (%)	0.70	0.76	1.2	0.54	1.8	0.45	0.14

Table 1. Raw data for multiple measurements of NU-901 and NU-100

Supplementary Note 3. Monte Carlo simulation of the average time spent at various separation distances

The considered kinetic processes can be illustrated as,

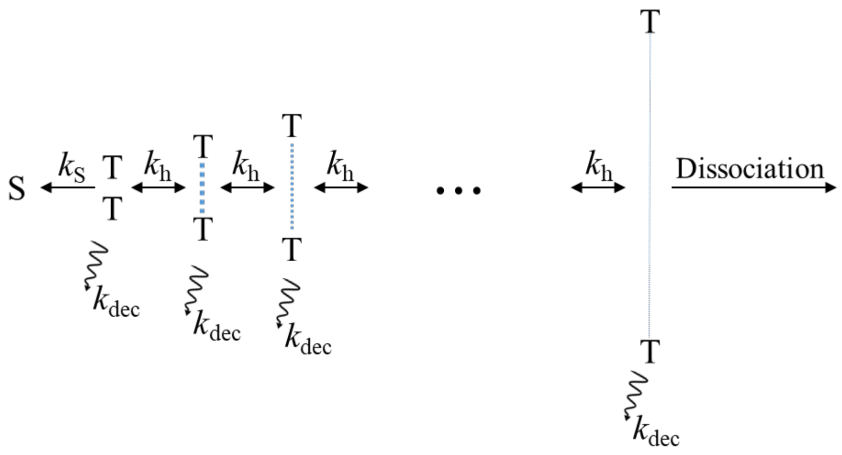

where k_h, k_S, k_{dec} are hopping rate, fusion rate, and spin relaxation rate. Note that it is depicted as 1D lattice for simplicity, but the calculations were done with 3D lattice parameters of NU-1000, NU-901, and anthracene crystals. Triplet pairs are allowed to fuse between the nearest-neighbors and they are set to dissociate beyond a specific separation. The initial condition was 3.8 nm separation, and it has a marginal effect on the results. Triplet pairs are assumed to fuse at the nearest-neighbor (NN) configuration and dissociate beyond 4 nm separation. We repeated the process until we collect 1000 trajectories of fusion, and averaged the time spent at each separation distance. The used parameters are summarized in Table 3, and the results are shown in Fig. S4.

Table 3. Model parameters used in the triplet-pair trajectory simulations. The NN and 2nd NN hopping distances of NU-1000 are 1 nm and 1.7 nm, respectively. The 2nd NN hopping includes both ab-plane and c-axis directions. Anthracene hopping rates are adopted from a reference [27] and the decoherence rate is based on the decay rates of quantum beating in tetracene [28]. For the hopping rates of MOF, we use the calculated values with PBE0 functional.

	Anthracene	NU-1000, NU-901
Hopping rates	4.1×10^{10} s^{-1} (*ab* plane)<br>1.4×10^{9} s^{-1} (*c* axis)	6.6×10^{9} s^{-1} (NN)<br>9.5×10^{6} s^{-1} (2nd NN)
Fusion rate	5×10^{9} s^{-1}	1×10^{9} s^{-1}
Spin relaxation rate	1×10^{8} s^{-1}	1×10^{8} s^{-1}
Dissociation distance	4 nm	4 nm

Supplementary Note 4. DFT calculation of hopping rates and exchange couplings

The hopping rates were calculated using Fermi's golden rule

$$k_{\text{hop}} = \frac{2\pi}{\hbar}\left|V_{\text{hop}}\right|^2 \rho_{\text{FC}} \tag{2}$$

where V_{hop} is the coupling that mediates the triplet hopping and ρ_{FC} is the Franck-Condon weighted density of states (FCWD). In the hopping integral, we include up to the second order, charge transfer (CT) terms, as they have been shown to be important[6,7]

$$V_{\text{hop}} = \langle DA^*|H|D^*A\rangle + \frac{\langle DA^*|H|D^-A^+\rangle\langle D^-A^+|H|D^*A\rangle}{E_{D^-A^+} - E_{D^*A}} + \frac{\langle DA^*|H|D^+A^-\rangle\langle D^+A^-|H|D^*A\rangle}{E_{D^+A^-} - E_{D^*A}} \tag{3}$$

The FCWD was approximated using Marcus theory[8,9]

$$\rho_{\text{FC}} = \frac{1}{\sqrt{4\pi\lambda_M k_B T}} \exp\left[-\frac{(\Delta E + \lambda_M)^2}{4\lambda_M k_B T}\right] \tag{4}$$

where ΔE is the energy gap and λ_M is the reorganization energy. Since the donor and the acceptor are symmetric, we have $\Delta E = 0$. Assuming that λ_M is dominated by intramolecular reorganization, we can write it in terms of the monomer properties

$$\lambda_M = \left(E_{\text{T}_1}[\mathbf{R}_{\text{S}_0}] - E_{\text{T}_1}[\mathbf{R}_{\text{T}_1}]\right) + \left(E_{\text{S}_0}[\mathbf{R}_{\text{T}_1}] - E_{\text{S}_0}[\mathbf{R}_{\text{S}_0}]\right) \tag{5}$$

where E_Ψ and $\mathbf{R}_\Psi$ are the energy and the geometry of the state Ψ, respectively.

The electronic structure calculations were performed using Q-Chem 5.1 software package.[10] We started with the structure of the NU-1000 MOF in the MOF Lab web module of Maxime Usdin.[11] In order to minimize the computational cost, we cropped just one or two organic linkers that were the most relevant to the problem: the monomer, the nearest neighbor dimer, and the second nearest neighbor dimer. The change imbalance was repaired by capping the carboxyl groups with hydrogen atoms. Then, a constrained geometry optimization was performed with the carboxyl oxygen atoms as the fixed atoms. The constraints ensured that the organic linkers did not move with respect to each other.

The hopping integral was calculated using constrained density functional theory (CDFT).[12] We used the ground state dimer geometries optimized using the B3LYP exchange-correlation functional[13,14] and the 6-31G* basis set.[15–18] A total of four diabatic states were calculated: $|\mathrm{D}^*A\rangle$, $|DA^*\rangle$, $|D^-\mathrm{A}^+\rangle$, and $|D^+\mathrm{A}^-\rangle$. Symmetric orthogonalization was performed in a pairwise manner to avoid the mixing of the excitonic states and the CT states. For example, we orthogonalized just the 2×2 matrix in the basis of $\{|D^*A\rangle, |D^-\mathrm{A}^+\rangle\}$ in calculating the coupling $\langle D^-A^+|H|D^*A\rangle$. The CDFT calculations were repeated on the same geometry with a range of functionals: CAM-B3LYP,[19] PBE,[20] PBE0,[21] LRC-ωPBEh,[22] M06-L,[23] and M06-2X.[24]

To calculate the reorganization energy, the monomer geometries were optimized in the S_0 and the T_1 states, and the S_0 and the T_1 energies were calculated at each geometry. We obtained the S_0 and the T_1 states using restricted and unrestricted density functional theory (RDFT and UDFT), respectively. The excited

state optimization and reorganization energy calculation were repeated with each of the abovementioned functionals.

The simulated reorganization energies, hopping integrals, and hopping rates are summarized in Table 1. Not surprisingly, the hopping rates exhibit a strong dependence on the fraction of exact exchange. Whereas PBE predicts the nearest neighbor hopping rate of 4.0×10^{10} s^{-1}, PBE0 predicts the rate to be almost two orders of magnitude smaller at 6.6×10^{9} s^{-1}. The decrease in the hopping rate is a combined effect of a decrease in the couplings and an increase in the reorganization energy. In addition, it is concerning that LRC-ωPBEh predicts the second nearest neighbor hopping integral to be zero. To be precise, the value is smaller than the convergence tolerance of the electronic structure calculations (10^{-11} au).

We suspect that the application of range-separated hybrids, such as CAM-B3LYP and LRC-ωPBEh, in conjunction with CDFT might be problematic. It has been shown that global hybrids, such as B3LYP and PBE0, can give more accurate excitation energies than range-separated hybrids when used in conjunction with ΔSCF.[25,26] In particular, range-separated hybrids tend to overestimate the energies. The reason might be that range-separated hybrids overcompensate for the delocalization error which orbital relaxation corrects to some extent. A similar situation arises in the case of CDFT, which is also a state-specific method. Then, we expect that range-separated hybrids would underestimate the couplings and overestimate the energies. Furthermore, B3LYP and PBE0 can be expected to give the most reliable estimates of the energetics.

Table 1. Simulated reorganization energies, FCWD, hopping integrals, and hopping rates. For rows with two functionals that are separated by a slash, the hopping rates have been calculated using the hopping integrals in the first functional and the reorganization energies in the second functional.

Functional	Reorganization Energy (eV)			FCWD (eV^{-1})	Dimer	Hopping Integral			Hopping Rate (s^{-1})
	S_0	T_1	Total			Direct (μeV)	HT (meV)	ET (meV)	
B3LYP	0.27	0.28	0.55	1.2×10^{-2}	NN	0.113	6.00	5.87	1.6×10^{10}
					2nd NN	0.000	0.25	0.26	2.9×10^{7}
CAM-B3LYP	0.44	0.45	0.88	3.4×10^{-4}	NN	0.002	1.22	1.18	1.9×10^{7}
					2nd NN	0.000	0.02	0.02	6.7×10^{3}
CAM-B3LYP / B3LYP					NN				6.4×10^{8}
					2nd NN				2.3×10^{5}
PBE	0.17	0.18	0.36	9.3×10^{-2}	NN	10.000	10.76	10.52	4.0×10^{11}
					2nd NN	0.000	0.60	0.60	1.3×10^{9}
PBE0	0.29	0.30	0.59	7.7×10^{-3}	NN	0.048	4.79	4.69	6.6×10^{9}
					2nd NN	0.000	0.18	0.18	9.5×10^{6}
LRC-ωPBEh	0.44	0.45	0.89	3.3×10^{-4}	NN	0.001	0.30	0.28	1.0×10^{6}
					2nd NN	0.000	0.00	0.00	6.9×10^{-7}
LRC-ωPBEh / PBE					NN				2.9×10^{8}
					2nd NN				2.0×10^{-4}

LRC-ωPBEh / PBE0					NN				2.4×10^{7}
					2nd NN				1.6×10^{-5}
M06-L	0.19	0.21	0.40	5.6×10^{-2}	NN	1.926	7.65	7.44	1.2×10^{11}
					2nd NN	0.000	0.29	0.28	1.7×10^{8}
M06-2X	0.40	0.42	0.82	6.6×10^{-2}	NN	0.006	1.66	1.61	6.7×10^{7}
					2nd NN	0.000	0.03	0.03	2.4×10^{4}
M06-2X / M06-L					NN				5.7×10^{9}
					2nd NN				2.0×10^{6}

The triplet-triplet exchange was estimated by the coupling

$$J\sim\langle m_1=+1, m_2=-1|H|m_1=-1, m_2=+1\rangle \quad (6)$$

where m_1 and m_2 are the spins of the two monomers. There is not a simple equivalence between the spin-1 particle wavefunction in the Heisenberg model and the spin-1/2 particle wavefunction in electronic structure theory. However, we expect that the above coupling would estimate the triplet-triplet exchange to the order of magnitude. The coupling itself was calculated using the same procedure as the hopping integrals.

The simulated couplings are summarized in Table 2. Like the case of the hopping integrals, CAM-B3LYP and LRC-ωPBEh predict the couplings to be zero within the tolerance. Again, we suspect that the application of range-separated hybrids in conjunction with CDFT might be leading to a gross underestimation of the coupling.

Table 2. Simulated couplings that approximate triplet-triplet exchange.

Functional	Exchange Coupling	
	NN (μeV)	2nd NN (μeV)
B3LYP	43.1	0.051
CAM-B3LYP	0.00	0.000
PBE	229	0.423
PBE0	18.4	0.017
LRC-ωPBEh	0.00	0.000
M06-L	83.2	0.102
M06-2X	1.97	0.000

Supplementary Note 5. Optimization of MOF design

As noted in the main text, the magnitude of the singlet-quintet resonance observed in NU-1000 is 2%. To realize the potential of singlet-quintet mixing, we expect that the tailorability of MOFs can be exploited to engineer larger resonances.

Figure S5 predicts trends in the magnitude of the singlet-quintet resonance as a function of three key parameters: the rate of TT pairs separating back into individual triplet excitons, k_{-1}; the formation rate of singlet excitons from TT pairs, k_S; and the zero-field splitting parameter, D, which measures the coupling of the singlet and quintet states. Three trends are observed.

(i) The resonance increases when we retard the separation of TT states back into individual triplets (Fig. S5(a))
(ii) The resonance increases when we increase the rate of singlet exciton formation from TT states (Fig. S5(b))
(iii) Increasing the coupling of singlet and quintet states increases the rate of spin mixing, which in turn can increase the magnitude of the resonance in the regime where $D < J$, where J is the exchange splitting (Fig. S5(c)).

The first two trends have a significant impact on the resonance and emphasize the potential importance of engineering the excitonic energy levels and employing exothermic fusion materials. When optimized, the peak resonance is observed in Fig. S5(d) to increase from 2% to 65%.

There are also potential improvements that may be obtained through structural design of the MOFs. Increasing the nearest neighbor distance by engineering reticular derivatives of NU-1000 is the most obvious target for future improvements. Further separating the pyrene cores by employing larger linker arms, for instance, is likely to render the exchange interaction negligible, such that spin mixing could occur even in the absence of a magnetic field. Too much separation, however, might slow down the fusion and hopping rate. The fusion rate has a quadratic dependence on triplet density, causing separation to impare the upconversion performance at low excitation intensity. Thus, the tradeoff between triplet diffusion and singlet-quintet resonant efficiency enhancements suggests optimization of localization may depend on the excitation intensity during operation.

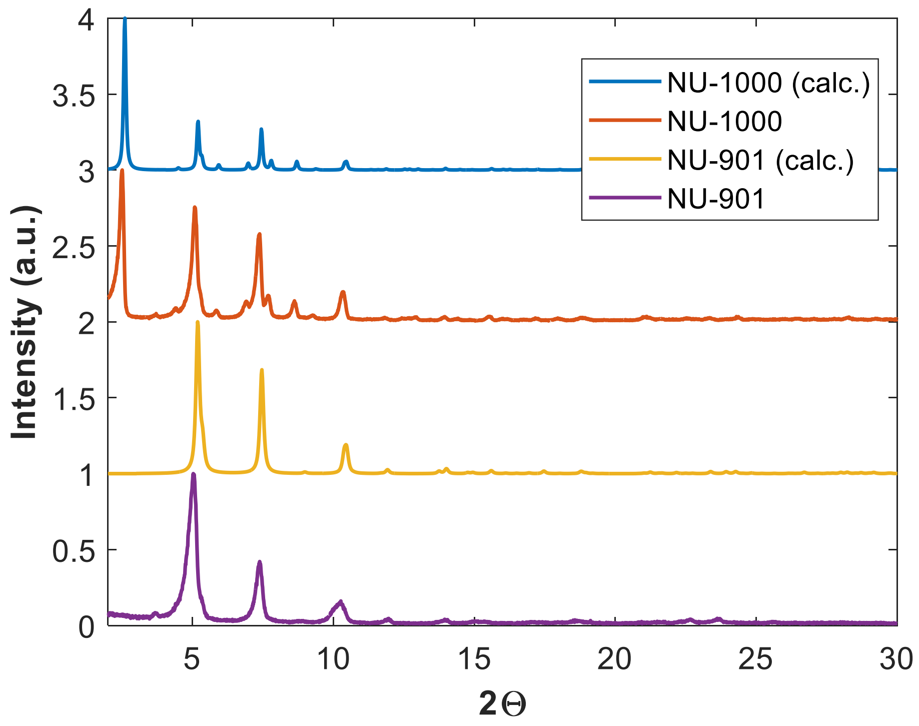

Fig. S1. Calculated and experimental powder X-ray diffraction pattern of NU-1000 and NU-901.

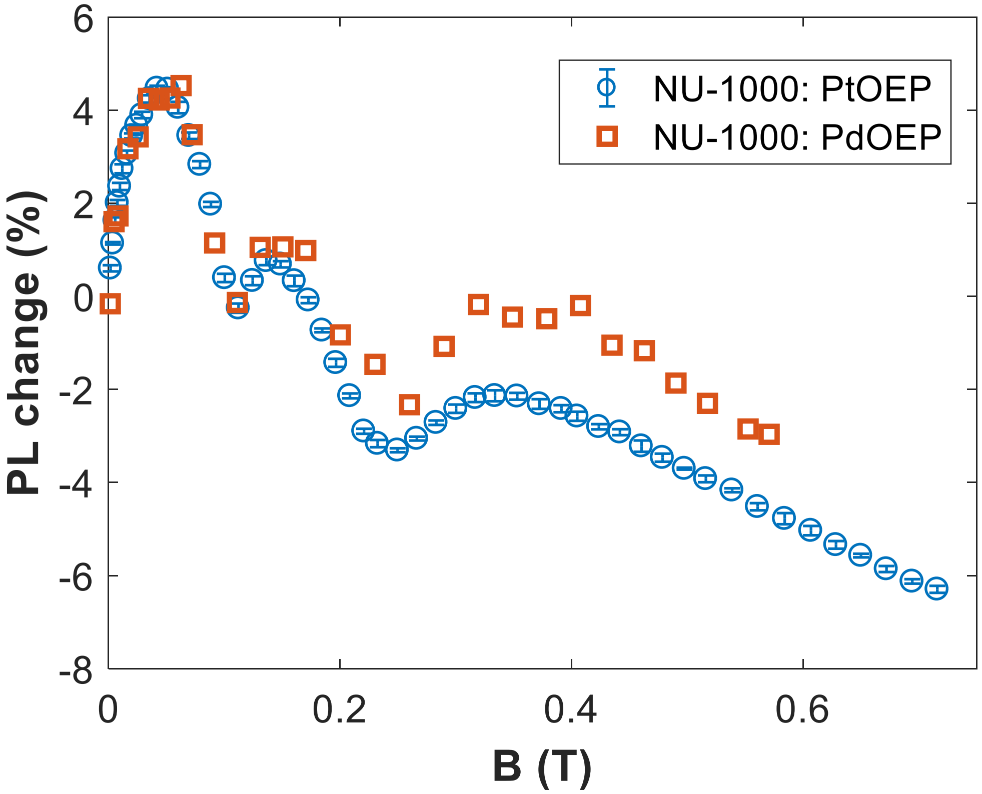

Fig. S2. Magnetic field effect on the unconverted emission of NU-1000: PdOEP and NU-1000: PtOEP. The triplet level of PdOEP is lower than PtOEP, and the triplet energy transfer to NU-1000 might not be efficient. The upconverted emission of NU-1000:PdOEP was weaker, so we used an intense laser (Verdi G18, Coherent) to measure the MFE. For NU-1000: PtOEP curve, error bars are the standard deviation of the mean, and they are averaged from 5 independent sweeps.

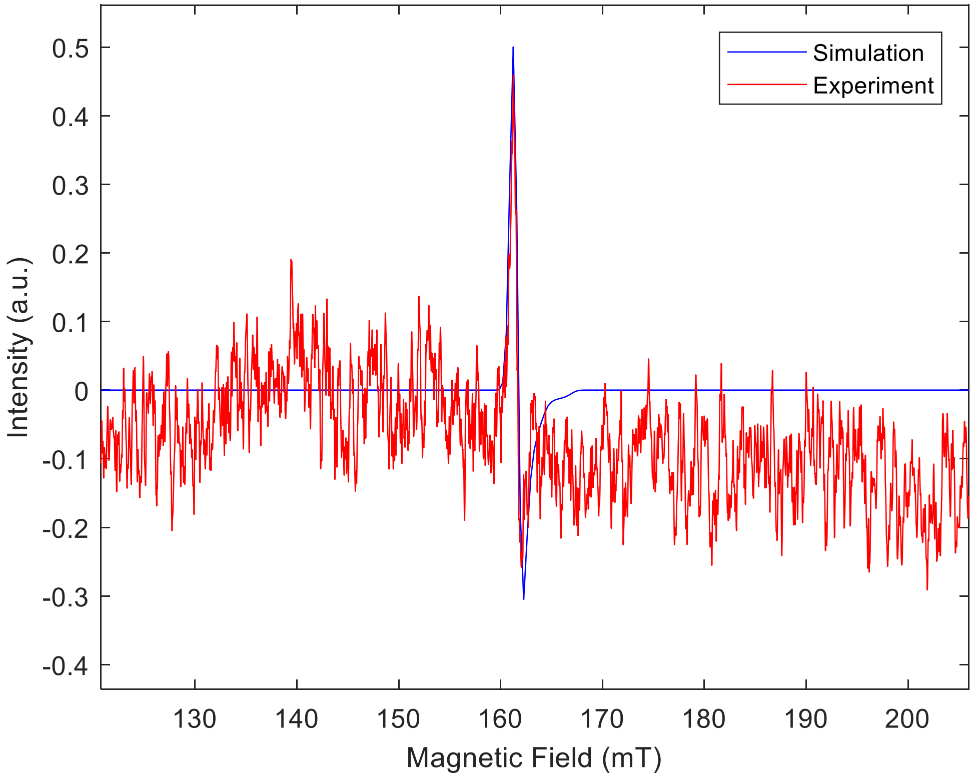

Fig. S3. Electron paramagnetic resonance (EPR) signal of NU-1000 and simulated curve with g=2.0. One characteristic feature for the EPR spectrum of a triplet state is the partially forbidden $|\Delta m_s| = 2$ transition that appears at g ~ 4.0 when the magnetic field orientation deviates from the principal axes of the molecule (known as a half-field transition)[29]. Such a signal is expected to be particularly pronounced for randomly oriented samples[8]. The apparent peak near 161.7 mT was assigned to be the half-field transition of the triplet exciton in the MOF. The peak position agrees with the simulation using |D| = 8.50 μeV and |E| = 1.17 μeV, further supporting the analysis we obtained from the MFE measurements. The g = 2 transitions were not observed probably because they were too broad to be separated from the baseline. The source of the broadening could be a distribution in the D and E values.

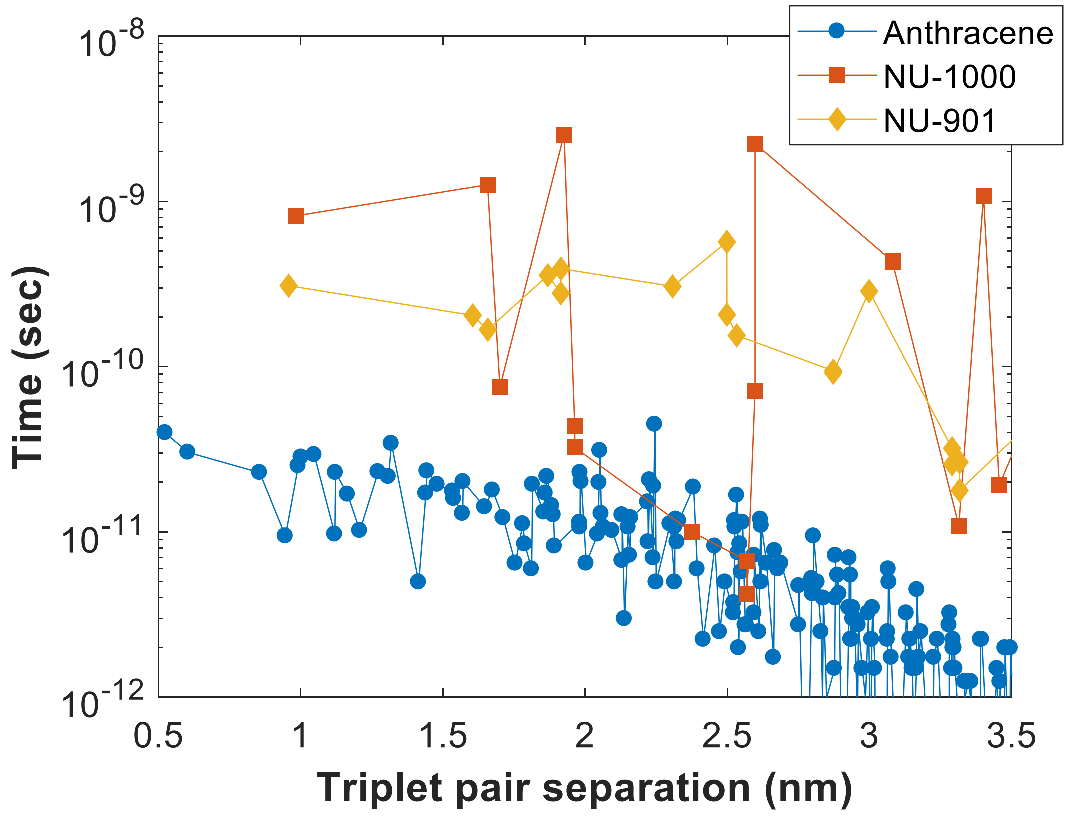

Fig. S4. The Monte Carlo simulations of the average time spent at various triplet pair separation distances. As expected, the average time spent at each site is much longer in NU-1000. The spin transition time is ~$\hbar\pi/D$ as shown in Fig. 1c. For D ~ 10 μeV, the transition time is ~200 ps. The triplet pairs in anthracene spend much shorter than the spin mixing time due to the fast hopping and dense structure. NU-1000, however, can hold a triplet pair long enough for the spin mixing. The triplet pairs in the denser MOF, NU-901, stay a shorter time at each site. The calculation results support the strong exchange features observed in NU-1000.

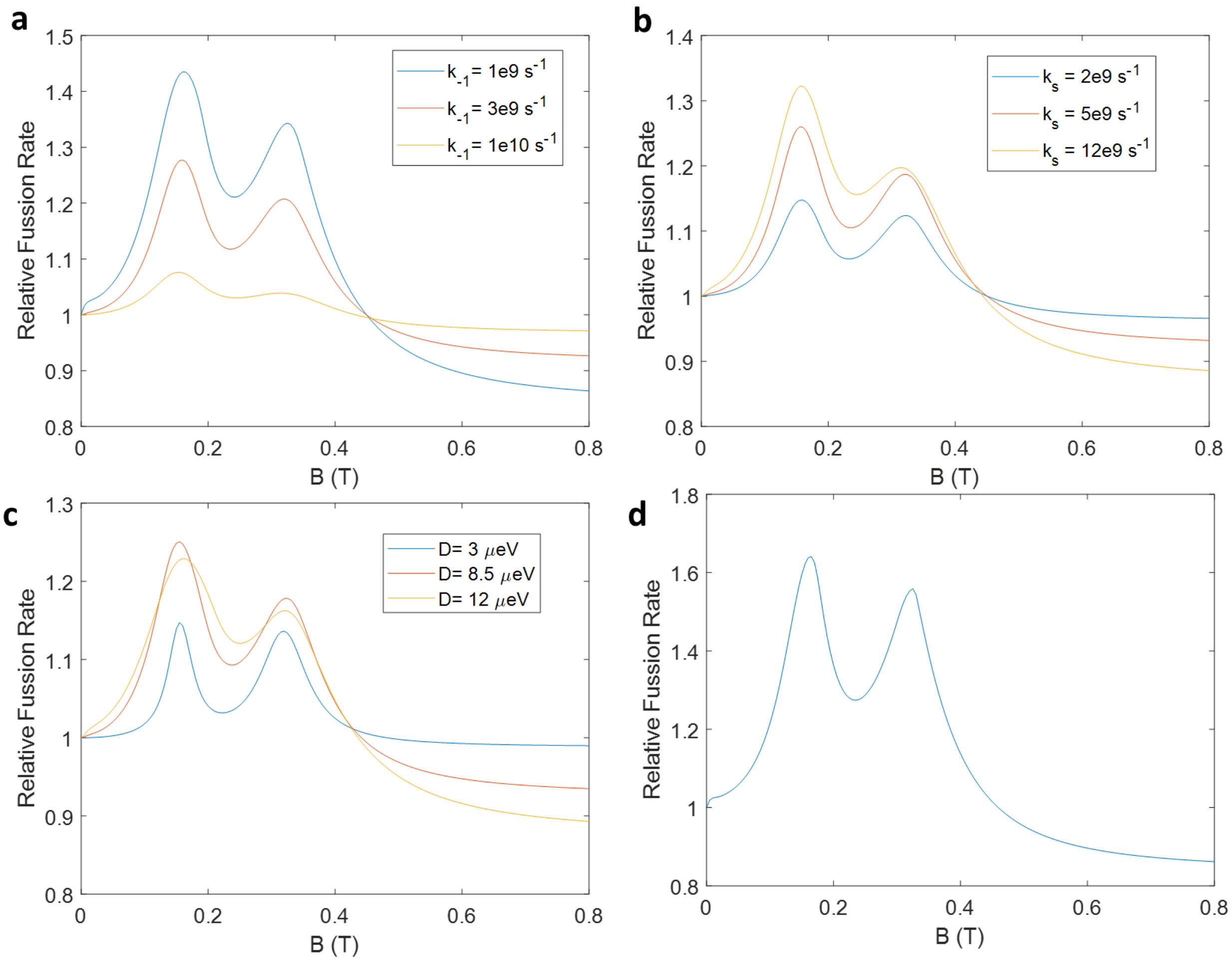

Fig. S5. Effect of kinetic parameters on the resonance peak intensity. We calculated the fusion rate with various kinetic parameters. The Johnson-Merrifield model was used with the parameters of k_S= 5×10^9 s^{-1} , k_{-1}= 3.3×10^9 s^{-1} , J= –6.1 μeV, D= 8.5 μeV and E= –1.1 μeV. Please note that the trend shown above may change with a different set of parameters. (a) Increased k_{-1} reduces the time for spin mixing, resulting in the decrease of the resonance peak. Fast hopping or fast spin relaxation can effectively increase k_{-1}. (b) An increase of k_s/k_{-1} will enhance the overall fusion efficiency. However, a large k_s will reduce the time for the spin mixing. (c) The zero-splitting parameter determines the coupling strength between the singlet and quintet states. The stronger coupling reduces the spin transition time, and the resonance peak increases. If D becomes dominant compared to J, the exchange feature will disappear. (d) Optimized MFE with k_S= 8×10^8 s^{-1} , k_{-1}= 5×10^7 s^{-1} , J= –6.1 μeV, D= 5 μeV and E= –0.8 μeV.

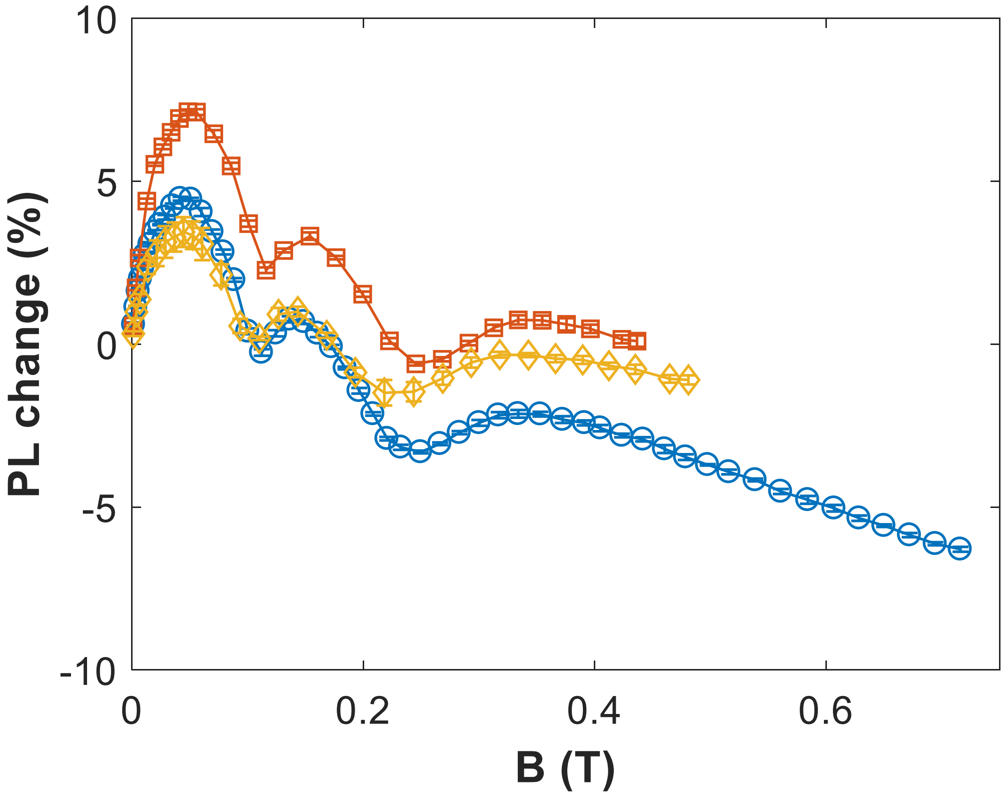

Fig. S6. MFE curves measured at different locations on a given sample of NU-1000 (red and blue), compared to a separate batch of NU-1000 with shorter PtOEP incorporation time (yellow). Possible explanations for the varying intensities include variations in PtOEP incorporation due to differing local morphologies and different triplet densities under similar excitation intensities. Nevertheless, the peak positions are identical. Error bars are the standard deviation of the mean, and they are averaged from 7 (red), 5 (blue), 4 (yellow) independent sweeps.

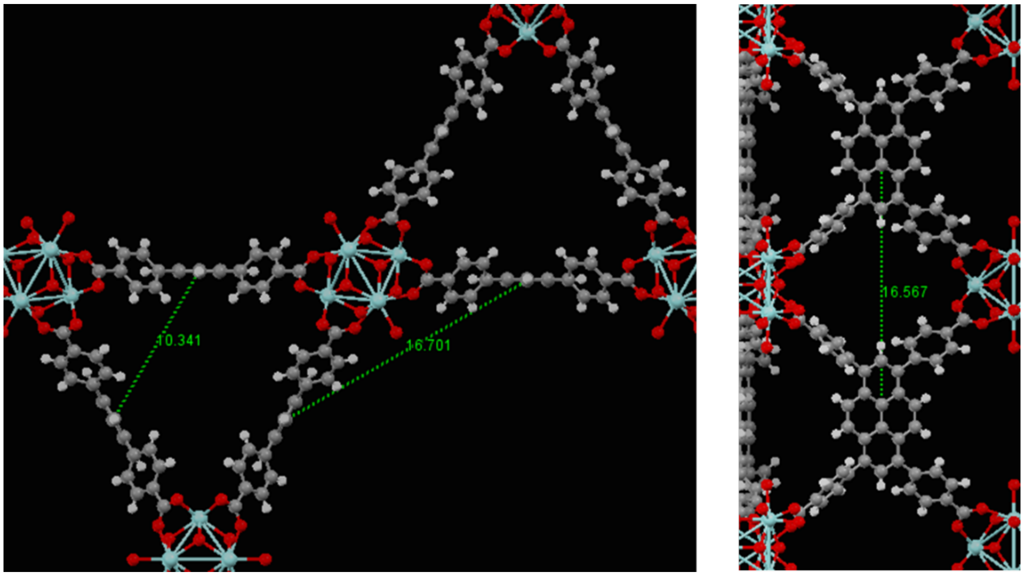

Fig. S7. Intermolecular distances in *ab* plane (left) and along *c* axis (right). The nearest neighbor distance is 1 nm and the second nearest neighbor distance is 1.7 nm.

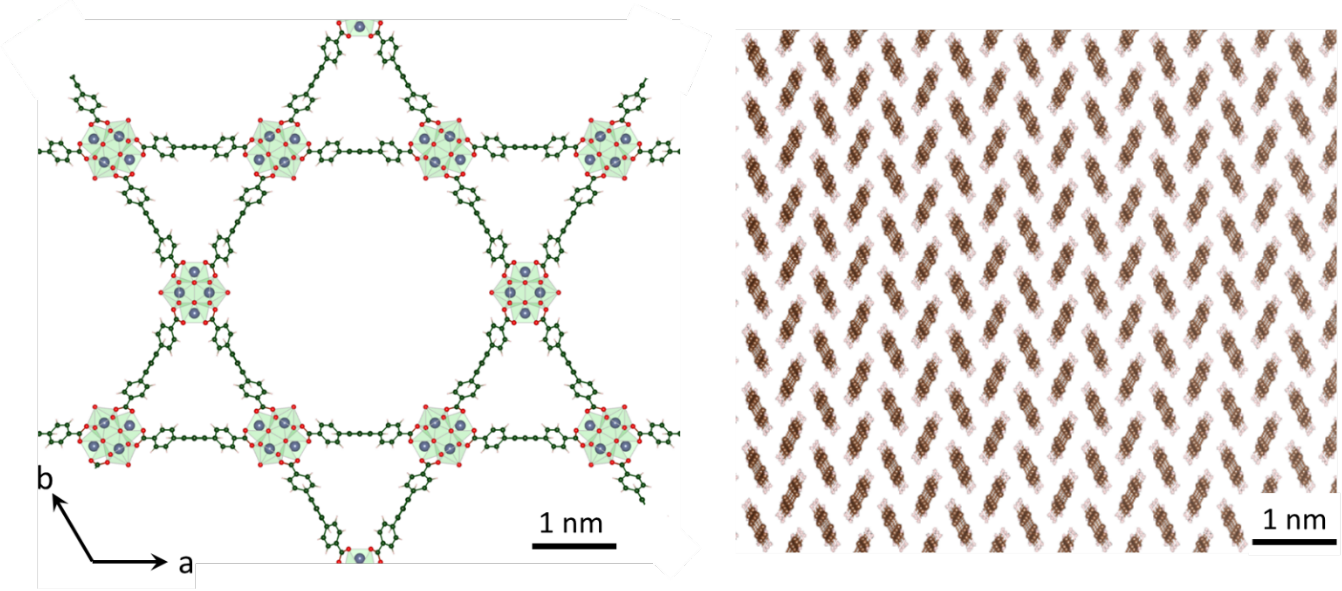

Fig. S8. Comparison of the *ab* plane of NU-1000 (left) and anthracene (right) on the same length scale. The molar volume of NU-1000 is 2220 cm^3, and anthracene is 143 cm^3. A TIPS-tetracene crystal[31], one of the bulkiest singlet fission materials, has a molar volume of 546 cm^3 that is still four times lower than NU-1000.

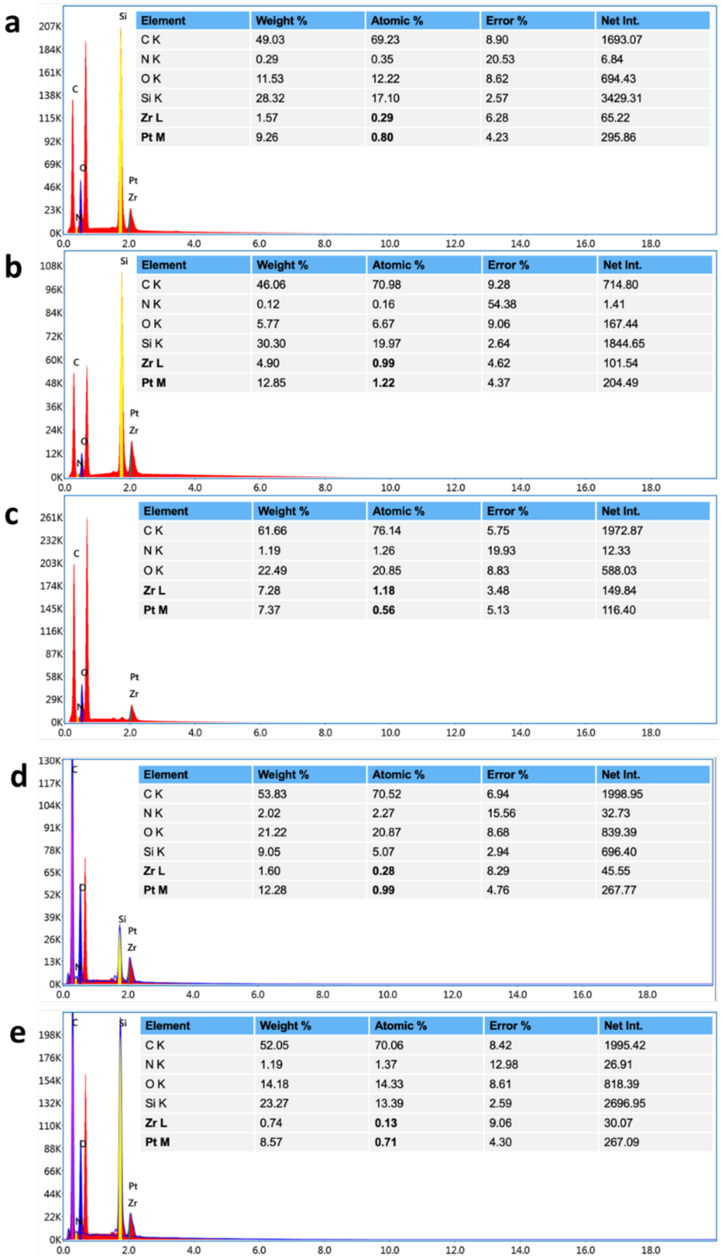

a

Element	Weight %	Atomic %	Error %	Net Int.
C K	49.03	69.23	8.90	1693.07
N K	0.29	0.35	20.53	6.84
O K	11.53	12.22	8.62	694.43
Si K	28.32	17.10	2.57	3429.31
**Zr L**	1.57	**0.29**	6.28	65.22
**Pt M**	9.26	**0.80**	4.23	295.86

b

Element	Weight %	Atomic %	Error %	Net Int.
C K	46.06	70.98	9.28	714.80
N K	0.12	0.16	54.38	1.41
O K	5.77	6.67	9.06	167.44
Si K	30.30	19.97	2.64	1844.65
**Zr L**	4.90	**0.99**	4.62	101.54
**Pt M**	12.85	**1.22**	4.37	204.49

c

Element	Weight %	Atomic %	Error %	Net Int.
C K	61.66	76.14	5.75	1972.87
N K	1.19	1.26	19.93	12.33
O K	22.49	20.85	8.83	588.03
**Zr L**	7.28	**1.18**	3.48	149.84
**Pt M**	7.37	**0.56**	5.13	116.40

d

Element	Weight %	Atomic %	Error %	Net Int.
C K	53.83	70.52	6.94	1998.95
N K	2.02	2.27	15.56	32.73
O K	21.22	20.87	8.68	839.39
Si K	9.05	5.07	2.94	696.40
**Zr L**	1.60	**0.28**	8.29	45.55
**Pt M**	12.28	**0.99**	4.76	267.77

e

Element	Weight %	Atomic %	Error %	Net Int.
C K	52.05	70.06	8.42	1995.42
N K	1.19	1.37	12.98	26.91
O K	14.18	14.33	8.61	818.39
Si K	23.27	13.39	2.59	2696.95
**Zr L**	0.74	**0.13**	9.06	30.07
**Pt M**	8.57	**0.71**	4.30	267.09

Fig. S9. Energy Dispersive Spectroscopy (EDS) elemental analysis of NU-1000:PtOEP (a~c) and NU-901:PtOEP (d~e) systems. The above figures show elemental mapping measurement results on different randomly chosen areas. The average atomic ratio of Pt/Zr is 1.5 ± 0.6 and 4.5 ± 0.7 for NU-1000:PtOEP and NU-901:PtOEP, respectively. Please note that the PtOEP washing step after the drop casting cannot ensure the complete removal of excess PtOEP, resulting in a higher measured Pt/Zr atomic ratio by EDS than the true value for the MOF:PtOEP systems. Further, the residual PtOEP also led to inhomogeneity of the samples, as reflected by varying Pt/Zr atomic ratios in different areas.

References

1. MERRIFIELD, R. E. Magnetic Effects on Triplet Exciton Interactions. *Pure Appl. Chem.* **27**, 481–498 (1971).
2. Johnson, R. C. & Merrifield, R. E. Effects of magnetic fields on the mutual annihilation of triplet excitons in anthracene crystals. *Phys. Rev. B* **1**, 896–902 (1970).
3. Yago, T., Ishikawa, K., Katoh, R. & Wakasa, M. Magnetic field effects on triplet pair generated by singlet fission in an organic crystal: Application of radical pair model to triplet pair. *J. Phys. Chem. C* **120**, 27858–27870 (2016).
4. He, L., Li, M., Urbas, A. & Hu, B. Magnetophotoluminescence line-shape narrowing through interactions between excited states in organic semiconducting materials. *Phys. Rev. B - Condens. Matter Mater. Phys.* **89**, 1–6 (2014).
5. Lin, T.-A., Perkinson, C. F. & Baldo, M. A. Strategies for High-Performance Solid-State Triplet–Triplet-Annihilation-Based Photon Upconversion. *Adv. Mater.* **32**, 1908175 (2020).
6. Olaya-Castro, A. & Scholes, G. D. Energy transfer from Förster-Dexter theory to quantum coherent light-harvesting. *Int. Rev. Phys. Chem.* **30**, 49–77 (2011).
7. Adams, M., Kozlowska, M., Baroni, N., Oldenburg, M., Ma, R., Busko, D., Turshatov, A., Emandi, G., Senge, M. O., Haldar, R., Wöll, C., Nienhaus, G. U., Richards, B. S. & Howard, I. A. Highly Efficient One-Dimensional Triplet Exciton Transport in a Palladium-Porphyrin-Based Surface-Anchored Metal-Organic Framework. *ACS Appl. Mater. Interfaces* **11**, 15688–15697 (2019).
8. Lluch, J. M. Perspective on 'on the theory of oxidation-reduction reactions involving electron transfer. I'. *Theor. Chem. Acc.* **103**, 231–233 (2000).
9. Marcus, R. A. Chemical and electrochemical electron-transfer theory. *Annu. Rev. Phys. Chem.* **15**, 155–196 (1964).
10. Shao, Y., Gan, Z., Epifanovsky, E., Gilbert, A. T. B., Wormit, M., Kussmann, J., Lange, A. W., Behn, A., Deng, J., Feng, X., Ghosh, D., Goldey, M., Horn, P. R., Jacobson, L. D., Kaliman, I. *et al.* Advances in molecular quantum chemistry contained in the Q-Chem 4 program package. *Mol. Phys.* **113**, 184–215 (2015).
11. Usdin, M., Chung, G. & Snurr, R. MOF Lab.
12. Kaduk, B., Kowalczyk, T. & Van Voorhis, T. Constrained density functional theory. *Chem. Rev.* **112**, 321–370 (2012).
13. Becke, A. D. Density-functional thermochemistry. III. The role of exact exchange. *J. Chem. Phys.* **98**, 5648–5652 (1993).
14. Stephens, P. J., Devlin, F. J., Chabalowski, C. F. & Frisch, M. J. Ab Initio calculation of vibrational absorption and circular dichroism spectra using density functional force fields. *J. Phys. Chem.* **98**, 11623–11627 (1994).
15. Hariharan, P. C. & Pople, J. A. The influence of polarization functions on molecular orbital hydrogenation energies. *Theor. Chim. Acta* **28**, 213–222 (1973).
16. Krishnan, R., Binkley, J. S., Seeger, R. & Pople, J. A. Self-consistent molecular orbital methods. XX. A basis set for correlated wave functions. *J. Chem. Phys.* **72**, 650–654 (1980).
17. Francl, M. M., Pietro, W. J., Hehre, W. J., Binkley, J. S., Gordon, M. S., DeFrees, D. J. & Pople, J. A. Self-consistent molecular orbital methods. XXIII. A polarization-type basis set for second-row elements. *J. Chem. Phys.* **77**, 3654–3665 (1982).
18. Clark, T., Chandrasekhar, J., Spitznagel, G. W. & Schleyer, P. V. R. Efficient diffuse function-augmented basis sets for anion calculations. III. The 3-21+G basis set for first-row elements, Li–F. *J. Comput. Chem.* **4**, 294–301 (1983).

19. Yanai, T., Tew, D. P. & Handy, N. C. A new hybrid exchange-correlation functional using the Coulomb-attenuating method (CAM-B3LYP). *Chem. Phys. Lett.* **393**, 51–57 (2004).
20. Perdew, J. P., Burke, K. & Ernzerhof, M. Generalized gradient approximation made simple. *Phys. Rev. Lett.* **77**, 3865–3868 (1996).
21. Adamo, C. & Barone, V. Toward reliable density functional methods without adjustable parameters: The PBE0 model. *J. Chem. Phys.* **110**, 6158–6170 (1999).
22. Rohrdanz, M. A., Martins, K. M. & Herbert, J. M. A long-range-corrected density functional that performs well for both ground-state properties and time-dependent density functional theory excitation energies, including charge-transfer excited states. *J. Chem. Phys.* **130**, 54112 (2009).
23. Zhao, Y. & Truhlar, D. G. A new local density functional for main-group thermochemistry, transition metal bonding, thermochemical kinetics, and noncovalent interactions. *J. Chem. Phys.* **125**, 194101 (2006).
24. Zhao, Y. & Truhlar, D. G. The M06 suite of density functionals for main group thermochemistry, thermochemical kinetics, noncovalent interactions, excited states, and transition elements: Two new functionals and systematic testing of four M06-class functionals and 12 other function. *Theor. Chem. Acc.* **120**, 215–241 (2008).
25. Kowalczyk, T., Tsuchimochi, T., Chen, P. T., Top, L. & Van Voorhis, T. Excitation energies and Stokes shifts from a restricted open-shell Kohn-Sham approach. *J. Chem. Phys.* **138**, (2013).
26. Hait, D., Zhu, T., McMahon, D. P. & Van Voorhis, T. Prediction of excited-state energies and singlet-triplet gaps of charge-transfer states using a restricted open-shell Kohn-Sham approach. *J. Chem. Theory Comput.* **12**, 3353–3359 (2016).
27. Suna, A. Kinetics of exciton-exciton annihilation in molecular crystals. *Phys. Rev. B* **1**, 1716 (1970).
28. Burdett, J. J. & Bardeen, C. J. Quantum beats in crystalline tetracene delayed fluorescence due to triplet pair coherences produced by direct singlet fission. *J. Am. Chem. Soc.* **134**, 8597–8607 (2012).
29. Wasserman, E., Snyder, L. C. & Yager, W. A. ESR of the Triplet States of Randomly Oriented Molecules. *J. Chem. Phys.* **41**, 1763–1772 (1964).
30. Goldfarb, D. & Stoll, S. *EPR Spectroscopy: Fundamentals and Methods*. (Wiley, 2018).
31. Xu, X., Shan, B., Kalytchuk, S., Xie, M., Yang, S., Liu, D., Kershaw, S. V. & Miao, Q. Synthesis, solution-processed thin film transistors and solid solutions of silylethynylated diazatetracenes. *Chem. Commun.* **50**, 12828–12831 (2014).
